# Radiative mixed convection flow of maxwell nanofluid over a stretching cylinder with joule heating and heat source/sink effects

**DOI:** 10.1038/s41598-020-74393-2

**Published:** 2020-10-20

**Authors:** Saeed Islam, Arshad Khan, Poom Kumam, Hussam Alrabaiah, Zahir Shah, Waris Khan, Muhammad Zubair, Muhammad Jawad

**Affiliations:** 1 Faculty of Mathematics & Statistics, Ton Duc Thang University, Ho Chi Minh City, 70000 Vietnam; 2 Informetrics Research Group, Ton Duc Thang University, Ho Chi Minh City, 70000 Vietnam; 3 College of Aeronautical Engineering, National University of Sciences and Technology (NUST), Sector H-12, Islamabad, 44000 Pakistan; 4 KMUTT Fixed Point Research Laboratory, Room SCL 802 Fixed Point Laboratory, Science Laboratory Building, Department of Mathematics, Faculty of Science, King Mongkut’s University of Technology Thonburi (KMUTT), 126 Pracha-Uthit Road, Bang Mod, Thrung Khru, Bangkok, 10140 Thailand; 5 Department of Medical Research, China Medical University Hospital, China Medical University, Taichung, 40402 Taiwan; 6 College of Engineering, Al Ain University, Al Ain, 64141 UAE; 7 Department of Mathematics, Tafila Technical University, Tafila, 66110 Jordan; 8 Department of Mathematics, University of Lakki Marwat, Lakki Marwat, 28420 Khyber Pakhtunkhwa Pakistan; 9 Department of Mathematics, Division of Science and Technology, University of Education Lahore, Lahore, 54770 Pakistan; 10 Department of Mathematics, Abdul Wali Khan University, Mardan, 23200 Khyber Pakhtunkhwa Pakistan

**Keywords:** Engineering, Mathematics and computing, Physics

## Abstract

This work analyses thermal effect for a mixed convection flow of Maxwell nanofluid spinning motion produced by rotating and bidirectional stretching cylinder. Impacts of Joule heating and internal heat source/sink are also taken into account for current investigation. Moreover, the flow is exposed to a uniform magnetic field with convective boundary conditions. The modeled equations are converted to set of ODEs through group of similar variables and are then solved by using semi analytical technique HAM. It is observed in this study that, velocity grows up with enhancing values of Maxwell, mixed convection parameters and reduces with growing values of magnetic parameter. Temperature jumps up with increasing values of heat source, Eckert number, Brownian motion,thermophoresis parameter and jumps down with growing values of Prandtl number and heat sink. The concentration is a growing function of thermophoresis parameter and a reducing function of Brownian motion and Schmidt number.

## Introduction

Recently, due to rapid development in modern industry, the scientists and researchers are forced to search such techniques and methods those are used for enhancement of heat transmission in heat exchanger equipment. In order to fulfill these requirements, scientists and researchers developed a new type of fluid, named as nanofluid and is used for commercial and industrial applications. This type of fluid is prepared by suspending nanoparticles in some base/pure fluid. Experiments have shown that combination of nanoparticles with base fluid enhances the coefficient of heat transmission of nanofluids. The usual materials used for nanoparticles are ${\text{Al}}_{2} {\text{O}}_{3} ,\,\,{\text{Cu}},\,\,{\text{TiO}}_{2} ,\,\,{\text{Ag}}$ etc.The quantity of nanoparticles was first suggested by Choi^1^ for augmenting thermal properties of pure fluid. Later, the subject of nanofluid wasdeveloped by a number of scientists and researchers. Elahi et al.^2^ examined mixed convection flow fornanoparticleson aporous surface by considering various shapes of nanoparticles. Dogonchi along with Ganji^3^ studied magnetic heat transfer for nanofluidpast a stretched surface and noticed an augmentation in flow characteristics with growing values of thermal radiations. These two authors^4^ have also discussed MHD nanofluid flow and transfer of heat by using Joule heating between two surfaces. The reader can further study about nanofluid in ref^5–8^.

For mixed convection flow,the difference of concentration and temperature results in buoyancy forces. These flows are considerable in various applications at industrial level. The collective effects of mass and heat transmission in mixed convection flows have extraordinary importance for complex engineering problems. Mukhopadhyay^9^ has discussed time-dependent mixed convection fluid flow with transmission of heatpasta a permeable stretched surface using slip condition. In this study, the author has solvedthe modeled problem numerically and has determined that, with augmentation of unsteadiness parameter there was a corresponding reduction in both temperature and velocity. Hayat et al.^10^ have examined mixed convection flow using convective boundary conditions past a stretched sheet. In this investigation, the authors have discussed numerical values for Nusselt number and skin-friction and have also compared their results with existing solutionsavailable in literature. Turkyilmazoglu^11^ hasanalytically studied solutions for mixed convection heat transmission of electrically conducted, viscoelastic fluid flowpast a stretchedsurface using Dufour and Soret effects. Shehzad et al.^12^ have discussed characteristics of heat and mass transmission for 3-D flow of Oldroyd-B fluid past a bi-directional stretchedsurfaceusing radiation effects. Xu and Pop^13^ have analyzed mixed convective flow for nanofluid past a stretched sheet using gyrotactic microorganisms and nanoparticles with uniform free stream.Moreover, tremendous investigation has also been carried out by Sankar et al.^14–19^ in the area of convective heat transfer by using various geometries and flow conditions. Their work has comprised of numerical as well as analytical investigations.

Study of heat transmission for linear and non-linear fluids plays very considerable role in various engineering processes for instance, electronic equipment cooling, extrusion process, inconservation of energy in nuclear reactor and cooling of nuclear reactor etc. Heat transport induced by rotating and stretching surfaces in visco-elastic fluid has a significant importance in plastic manufacturing because the final products quality is mainly dependent on heat transport. Numerous investigations have been carried out by the researchers for prediction of heat transport in flow of fluidfor rotating and stretching surfaces. Mustafa et al.^20^ have studied transmission of mass and heatamid two plates. They have examined in this study that, augmentation in magnitude of Schmidt number enhances the values of local Sherwood number while reduces the concentration profile. Kumar and Nath^21^ have carried out the analytical study of time dependent 3D MHD boundary layer flow and heat transmission for stretched plane surface. In this study, the authors have showed that analytical series solution is very much precise in the complete domain for all values of time. Alizadeh et al.^22^ have discussed MHD micropolar flow of fluid in a conduit filled with nanoparticles using thermal effects. Ashorynejad et al.^23^ have investigated heat transmission of MHD nanofluid past a stretching cylinder. In this investigation, the authors have solved the system of modeled ODEs by RK-4 method. Seth et al.^24,25^ have investigated Casson fluid flow with Newtonian heating and thermo diffusion effects using porous and non-Darcy porous media. Tripathi et al.^26^ have discussed double diffusive flow for hydromagneticnanofluid through a rotating channel using Hall Effect and viscous dissipation. Arifuzzaman et al.^27^ have discussed transmission of heat and mass for MHD fourth-grade radiative fluid flow over a porous plate using chemical reaction.

Fluid flow over a stretching flat plate or cylinder has achieved consideration from numerous researchers because of its significant applications at industrial level, such as liquid film for condensation procedure, growing of crystals, foods and papers manufacturing, glass fabrication and polymer extrusion etc. Due to its importance, many researchers diverted their attention towards the flow past a stretching flat plate or cylinder. Crane^28^ has studied the flow past a stretched sheet. Wang and Ng^29^ have discussed slip flow past a stretched cylinder. In this study, they have used suitable set of similarity transformation to transform the governing PDEs to set of non-linear ODEs. They have further transformed this set of non-linear ODEs to a simple form by using compressed variable and then solved this new system by using numerical integration. The main outcome of their study was that, they have determined that the magnitudes of shear stress and velocities are greatly trimmed down by slip flow. Bhattacharyya et al.^30^ havediscussedsimulation of Cattaneo-Christov heat flux for single as well as multi walled CNTs (Corbon Nanotubes) between two stretched rotating coaxial disks. Seth et al.^31^ studied entropy generation for flow of hydromagneticnanofluidover non-linear stretched surface using Navier’s velocity slip with convective heat transfer. The readers can further study about stretching flows by using different geometries in ref^32–37^.

In this work we shall endeavor to

i. Discuss mixed convection flow for Maxwell nanofluid with transfer of thermal energy over a stretching and rotating cylinder.
ii. Analyze heat transmission using Joule heating with heat generation/absorption in nanofluid flow.

The modeled problem will then be transformed to set of ODEs employing group of similar variables. The resultant set of ODEs will be solved by using HAM^38–40^. The behavior of different substantial parameters will be examined and discussed graphically. Moreover, a comparison will also be carried out for validation of current work with the results as available in literatur^46^.

## Physical and mathematical model

In this section we shall firstgive physical description of the problem. Then the mathematical formulation of problem and suitable set of dimensionless variables will be introduced. Moreover, some physical parameters will also be defined along with mathematical representations in this section.

### Physical description

**Figure 1:**
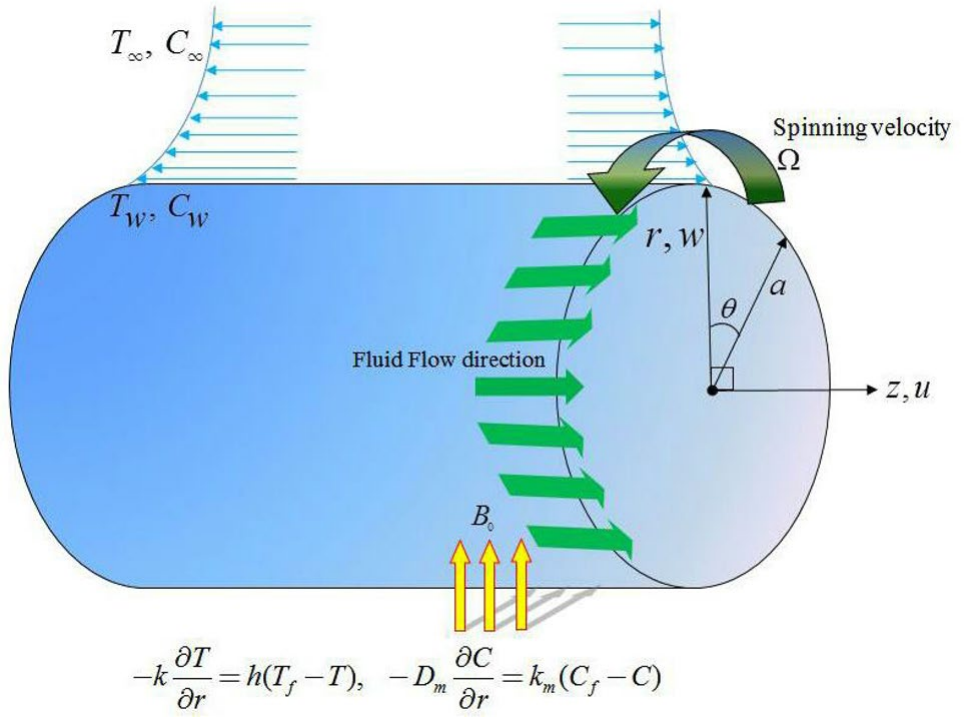
Geometry of flow problem.

For current flow problem the following assumptions are considered

i. Take a mixed convection flow for Maxwell nanofluid over a stretching and rotating cylinder of radius $a$.
ii. Magnetic field of intensity $B_{0}$ is applied to flow system.
iii. Let $V = \left[ {u,\,v,\,w} \right]$ be velocity field with $u,\,v$ and $w$ as its components along $z,\,\theta$ and $r$-axes respectively (See Fig. 1). Axis of cylinder is taken along z-axis and radial direction is along r-axis.
iv. Maxwell nanofluid model along with thermophoresis and Brownian motion effects are considered for flow problem.
v. Temperature and concentration at surface of cylinder aretaken as $T_{w} ,\,C_{w}$ while these quantities at free stream are $T_{\infty } ,\,C_{\infty }$. In concentration equation the chemical reaction is assumed to be overlooked.

### Mathematical description

Considering all assumptions as stated in “Physical description” section the mathematical representation for flow system takes the form^41–45^

1 $$\frac{\partial u}{{\partial z}} + \,\,\frac{w}{r}\,\, + \,\,\frac{\partial w}{{\partial r}}\,\, = \,\,0$$

2 $$\begin{gathered} u\frac{\partial u}{{\partial z}}\,\, + \,\,\,w\frac{\partial u}{{\partial r}}\,\,\, + \,\,\,\lambda_{1} \left[ {w^{2} \frac{{\partial^{2} u}}{{\partial r^{2} }}\,\,\, + \,\,\,2uw\frac{{\partial^{2} u}}{\partial r\partial z}\,\,\, + \,\,u^{2} \frac{{\partial^{2} u}}{{\partial z^{2} }}} \right] = - \frac{1}{\rho }\frac{\partial p}{{\partial z}}\,\,\, + \,\,\,\upsilon \left[ {\frac{1}{r}\frac{\partial u}{{\partial r}}\,\,\, + \,\,\,\frac{{\partial^{2} u}}{{\partial r^{2} }}} \right] \hfill \\ \,\,\,\,\,\,\,\,\,\,\, - \frac{{\sigma B_{0}^{2} }}{\rho }\left( {\lambda_{1} w\frac{\partial u}{{\partial r}}\,\, + \,\,u} \right) \, \,{ + }g\left[ {\frac{{\rho^{ * } - \rho }}{\rho }\left( {C - C_{\infty } } \right)\,\,\, + \,\,\,\beta_{T} \left( {T - T_{\infty } } \right)\left( {1 - C_{\infty } } \right)} \right]{, } \hfill \\ \end{gathered}$$

3 $$\begin{gathered} w\frac{\partial T}{{\partial r}}\,\,\, + \,\,\,u\frac{\partial T}{{\partial z}} = \alpha_{1} \left[ {\frac{1}{r}\frac{\partial T}{{\partial r}}\,\,\, + \,\,\,\frac{{\partial^{2} T}}{{\partial r^{2} }}} \right] + \tau \left[ {\frac{{D_{T} }}{{T_{\infty } }}\left( {\frac{\partial T}{{\partial r}}} \right)^{2} \,\,\, + \,\,\,D_{B} \frac{\partial T}{{\partial r}}\,\,\frac{\partial C}{{\partial r}}} \right]\, \hfill \\ \,\,\,\,\,\,\,\,\,\,\,\,\,\,\,\,\,\,\,\,\,\,\,\,\,\,\,\,\,\,\, + \frac{{Q_{0} }}{{\rho c_{p} }}\left( {T - T_{\infty } } \right)\,\,\, + \,\,\frac{{\sigma B_{0}^{2} }}{{\rho c_{p} }}\left( {v^{2} \,\,\, + \,\,\,u^{2} } \right) \hfill \\ \end{gathered}$$

4 $$w\frac{\partial C}{{\partial r}}\,\,\, + \,\,\,u\frac{\partial C}{{\partial z}} = D_{B} \left[ {\frac{{\partial^{2} C}}{{\partial r^{2} }}\,\,\, + \,\,\,\frac{1}{r}\frac{\partial C}{{\partial r}}\,\,} \right] + \frac{{D_{T} }}{{T_{\infty } }}\left[ {\frac{1}{r}\frac{\partial T}{{\partial r}}\,\, + \,\,\,\frac{{\partial^{2} T}}{{\partial r^{2} }}} \right]$$

Subjected BCs are

5 $$\begin{gathered} u = U = \frac{{U_{0} z}}{l},\,\,w = 0,\,\, - k\frac{\partial T}{{\partial r}}\,\,\, = \,\,\,h\,\left( {T_{f} \,\,\, - \,\,\,T} \right),\, - Dm\,\,\,\frac{\partial C}{{\partial r}}\,\, = \,\,k_{m} \left( {C_{f} \,\,\, - \,\,\,C} \right)\,at\,\,r = a \hfill \\ \hfill \\ C \to C_{\infty } ,\,\,u \to 0,\,\,T \to T_{\infty } \,\,\,\,\,\,as\,\,\,\,r \to \infty \hfill \\ \end{gathered}$$

Group of dimensionless variables is

6 $$\psi = \left( {U\upsilon z} \right)^{1/2} af\left( \eta \right),\,\,\,\,\phi \left( \eta \right) = \frac{{C - C_{\infty } }}{{C_{f} - C_{\infty } }}\,\,,\,\,\,\theta \left( \eta \right) = \frac{{T - T_{\infty } }}{{T_{f} - T_{\infty } }},\,\,\,\,\,\,\eta = \frac{{\left( {r^{2} - a^{2} } \right)}}{2a}\sqrt {\frac{U}{\upsilon z}}$$

Making use of Eq. (6) in Eqs. (1–5) we have

7 $$\begin{aligned} & \left( {2\gamma \eta \,\, + \,\,\,1} \right)f^{\prime\prime\prime}\,\, + \,\,ff^{\prime\prime}\,\, + \,\,\,2\gamma f^{\prime\prime} - f^{{\prime}{2}} - \beta_{1} {\text{Re}} \left( {2f^{2} f^{\prime\prime\prime}\,\,\, + \,\,\,\frac{{f^{2} f^{\prime\prime}}}{\eta }\,\,\, - \,\,\,4ff^{\prime}f^{\prime\prime}} \right) \hfill \\ & \quad + \lambda \left( {\theta \,\, + \,\,N_{r} \phi } \right) - M\left( {f^{\prime}\,\, - \,\,\beta_{1} ff^{\prime\prime}} \right) = 0 \hfill \\ \end{aligned}$$

8 $$\begin{aligned} & \left( {2\gamma \eta \,\, + \,\,1} \right)\theta^{\prime\prime} + {\text{PrN}}_{b} \left( {2\gamma \eta \,\, + \,\,1} \right)\theta^{\prime}\phi^{\prime} + \Pr f\theta^{\prime} + \Pr M\left( {Ec_{1} f^{{\prime}{2}} + Ec_{2} g} \right) \hfill \\ & \quad + \Pr N_{t} \left( {2\gamma \eta \,\, + \,\,1} \right)\theta^{{\prime}{2}} + \delta \Pr {\text{Re}} \theta = 0 \hfill \\ \end{aligned}$$

9 $$\left( {1 + 2\gamma \eta } \right)\phi^{\prime\prime} + 2\gamma \phi^{\prime} + Scf\phi^{\prime} + \frac{{N_{t} }}{{N_{b} }}\left[ {\left( {1 + 2\gamma \eta } \right)\theta^{\prime\prime} + 2\gamma \theta^{\prime}} \right] = 0$$

Subjected BCs are now

10 $$\begin{gathered} f\left( 0 \right) = 0,\,\,\,f^{\prime}\left( 0 \right) = 1,\,\,\,\,\,\theta^{\prime}\left( 0 \right) = - \gamma_{1} \left[ {1 - \theta \left( 0 \right)} \right],\,\,\,\phi^{\prime}\left( 0 \right) = - \gamma_{2} \left[ {1 - \phi \left( 0 \right)} \right], \hfill \\ f^{\prime}\left( \infty \right) = 0,\,\,\,\,\theta \left( \infty \right) = 0,\,\,\phi \left( \infty \right) = 0 \hfill \\ \end{gathered}$$

In above equations we have $\gamma = \left( {\frac{\upsilon l}{{U_{0} a^{2} }}} \right)^{1/2} =$ curvature parameter, $N_{r} = \frac{{\left( {\rho^{*} - \rho } \right)\Delta C}}{{\rho \beta_{T} \Delta T\left( {1 - \phi_{\infty } } \right)}} =$ Buoyancy ratio, $M = \frac{{\sigma B_{0}^{2} l}}{{\rho U_{0} }} =$ Hartman number, $N_{b} = \frac{{\tau D_{B} \Delta C}}{\upsilon } =$ Brownian motion parameter, $\Pr = \frac{\upsilon }{\alpha } =$ Prandtl number, $\lambda = \frac{{gl^{2} \beta_{T} \left( {1 - C_{\infty } } \right)\Delta T}}{{U_{0}^{2} z}} =$ Mixed convection parameter, $Sc = \frac{\upsilon }{{D_{B} }} =$ Schmidt number, $N_{t} = \frac{{\tau D_{T} \Delta T}}{{T_{\infty } \upsilon }} =$ Thermophoresis parameter, $Ec_{1} = \frac{{u_{w}^{2} }}{{c_{p} \Delta T}}$ = Eckert number for cylinder’s stretching, $Ec_{2} = \frac{{v_{w}^{2} }}{{c_{p} \Delta T}}$ = Eckert number for cylinder’s rotation, $\beta_{1} = \lambda_{1} a =$ Maxwell parameter and $\delta = \frac{{Q_{0} }}{{\rho c_{p} a}} =$ Heat generation/absorption parameter.

### Some quantities of importance

For modeled problem Nusselt and Sherwood numbers are given by

11 $$Nu_{z} = \frac{{zq_{w} }}{{k\left( {T_{f} - T_{\infty } } \right)}},\,\,\,\,\,\,\,\,\,Sh_{z} = \frac{{zh_{m} }}{{D_{B} \left( {C_{f} - C_{\infty } } \right)}}$$

In Eq. (11) $h_{m}$ and $q_{w}$ are defined as

12 $$\,h_{m} = \left( { - D_{B} \frac{\partial C}{{\partial r}}} \right)_{r = a} \,\,\,\,,\,\,\,\,\,\,\,\,q_{w} = \left( { - k\frac{\partial T}{{\partial r}}} \right)_{r = a}$$

Making use of Eq. (6) in Eq. (11) we have

13 $$Nu_{z} \left( {{\text{Re}}_{z} } \right)^{ - 1/2} = - \theta^{\prime}\left( 0 \right),\,\,\,\,\,\,\,\,\,Sh_{z} \left( {{\text{Re}}_{z} } \right)^{ - 1/2} = - \phi^{\prime}\left( 0 \right)$$

## Solution of problem

In current work **s**emi analytical method HAM determines solution for resultant set of ODEs as given in Eqs. (7–9) by applying the boundary conditions as stated in Eq. (10). The initial guess for the specified equations is stated below

14 $$f_{0} = 1 - e^{ - \eta } ,\,\,\,\Theta_{0} = \frac{{\gamma_{1} }}{{1 + \gamma_{1} }}e^{ - \eta } \,\,,\,\,\,\Phi_{0} \left( \eta \right) = \frac{{\gamma_{2} }}{{1 + \gamma_{2} }}e^{ - \eta }$$

The linear operators are stated as follows

15 $$L_{f} \left( {\,\,f\,\,} \right) = f^{\prime\prime\prime} - f^{\prime},\,\,\,L_{\Theta } \left( {\,\Theta \,} \right) = \theta^{\prime\prime} - \theta ,\,\,L_{\Phi } \left( \Phi \right) = \phi^{\prime\prime} - \phi$$

16 $$\begin{gathered} L_{{\overset{\lower0.5em\hbox{$\smash{\scriptscriptstyle\frown}$}}{f} }} (e_{1} \,\,\, + \,\,\,e_{2} e^{\eta } \,\, + \,\,e_{3} e^{ - \eta } ) = 0, \hfill \\ {\text{With}}\;\;\;\;{\text{L}}_{{\overset{\lower0.5em\hbox{$\smash{\scriptscriptstyle\frown}$}}{\theta } }} (e_{4} e^{\,\eta } \,\, + \,\,\,e_{4} e^{ - \eta } ) = 0, \hfill \\ {\text{L}}_{{\overset{\lower0.5em\hbox{$\smash{\scriptscriptstyle\frown}$}}{\phi } }} (e_{6} e^{\eta } \,\,\, + \,\,\,e_{7} e^{ - \eta } ) = 0 \, \hfill \\ \end{gathered}$$

In Eq. (16) ${\text{e}}_{i} \left( {for\,\,i = 1,\,2, \ldots ,7} \right)$ are constants.

Further

17 $$\begin{aligned} \, {\rm N}_{{\overset{\lower0.5em\hbox{$\smash{\scriptscriptstyle\frown}$}}{f} }} \, \left[ {\overset{\lower0.5em\hbox{$\smash{\scriptscriptstyle\frown}$}}{f} (\eta ;\zeta ),\overset{\lower0.5em\hbox{$\smash{\scriptscriptstyle\frown}$}}{\theta } (\eta ;\zeta ),\overset{\lower0.5em\hbox{$\smash{\scriptscriptstyle\frown}$}}{\phi } (\eta ;\zeta )} \right] & = \left( {2\gamma \eta \,\, + \,\,1} \right)\overset{\lower0.5em\hbox{$\smash{\scriptscriptstyle\frown}$}}{f}_{\eta \eta \eta } + 2\gamma \overset{\lower0.5em\hbox{$\smash{\scriptscriptstyle\frown}$}}{f}_{\eta \eta } + \overset{\lower0.5em\hbox{$\smash{\scriptscriptstyle\frown}$}}{f} \overset{\lower0.5em\hbox{$\smash{\scriptscriptstyle\frown}$}}{f}_{\eta \eta } - \overset{\lower0.5em\hbox{$\smash{\scriptscriptstyle\frown}$}}{f}_{\eta }^{2} \hfill \\ & \quad - \beta_{1} {\text{Re}} \left( {\frac{{\overset{\lower0.5em\hbox{$\smash{\scriptscriptstyle\frown}$}}{f}^{2} \overset{\lower0.5em\hbox{$\smash{\scriptscriptstyle\frown}$}}{f}_{\eta \eta } }}{\eta } + 2\overset{\lower0.5em\hbox{$\smash{\scriptscriptstyle\frown}$}}{f}^{2} \overset{\lower0.5em\hbox{$\smash{\scriptscriptstyle\frown}$}}{f}_{\eta \eta \eta } - 4\overset{\lower0.5em\hbox{$\smash{\scriptscriptstyle\frown}$}}{f} \overset{\lower0.5em\hbox{$\smash{\scriptscriptstyle\frown}$}}{f}_{\eta } \overset{\lower0.5em\hbox{$\smash{\scriptscriptstyle\frown}$}}{f}_{\eta \eta } } \right) - \,M\left( {\overset{\lower0.5em\hbox{$\smash{\scriptscriptstyle\frown}$}}{f}_{\eta } - \beta_{1} \overset{\lower0.5em\hbox{$\smash{\scriptscriptstyle\frown}$}}{f} \overset{\lower0.5em\hbox{$\smash{\scriptscriptstyle\frown}$}}{f}_{\eta \eta } } \right) + \lambda \left( {\overset{\lower0.5em\hbox{$\smash{\scriptscriptstyle\frown}$}}{\theta }_{\xi } + N_{r} \overset{\lower0.5em\hbox{$\smash{\scriptscriptstyle\frown}$}}{\phi } } \right) \hfill \\ \end{aligned}$$

18 $$\begin{gathered} {\rm N}_{{\overset{\lower0.5em\hbox{$\smash{\scriptscriptstyle\frown}$}}{\theta } }} \left[ {\overset{\lower0.5em\hbox{$\smash{\scriptscriptstyle\frown}$}}{f} (\eta ;\zeta ),\,\,\overset{\lower0.5em\hbox{$\smash{\scriptscriptstyle\frown}$}}{\theta } (\eta ;\zeta ),\,\,\,\overset{\lower0.5em\hbox{$\smash{\scriptscriptstyle\frown}$}}{\phi } (\eta ;\zeta )} \right] = \left( {2\gamma \eta \,\, + \,\,1} \right)\overset{\lower0.5em\hbox{$\smash{\scriptscriptstyle\frown}$}}{\theta }_{\eta \eta } + \Pr \overset{\lower0.5em\hbox{$\smash{\scriptscriptstyle\frown}$}}{f} \overset{\lower0.5em\hbox{$\smash{\scriptscriptstyle\frown}$}}{\theta }_{\eta } + \Pr \,N_{b} \left( {2\gamma \eta \,\, + \,\,1} \right)\overset{\lower0.5em\hbox{$\smash{\scriptscriptstyle\frown}$}}{\phi }_{\eta } \overset{\lower0.5em\hbox{$\smash{\scriptscriptstyle\frown}$}}{\theta }_{\eta } \hfill \\ + \Pr M\left( {Ec_{1} \overset{\lower0.5em\hbox{$\smash{\scriptscriptstyle\frown}$}}{f}_{\eta }^{2} \,\,\, + \,\,\,Ec_{2} \overset{\lower0.5em\hbox{$\smash{\scriptscriptstyle\frown}$}}{g} } \right)\, + \Pr N_{t} \left( {2\gamma \eta \,\, + \,\,1} \right)\overset{\lower0.5em\hbox{$\smash{\scriptscriptstyle\frown}$}}{\theta }_{{^{\eta } }}^{2} \,\, + \,\,\delta \Pr {\text{Re}} \overset{\lower0.5em\hbox{$\smash{\scriptscriptstyle\frown}$}}{\theta } \hfill \\ \,\,\,\,\,\,\,\,\,\,\,\,\,\,\,\,\,\,\,\,\,\,\,\,\,\,\,\,\,\,\,\,\,\,\,\,\,\,\,\,\,\,\,\,\,\,\,\,\,\,\,\,\,\,\,\,\,\,\,\,\,\,\,\,\,\,\,\,\,\, \hfill \\ \end{gathered}$$

19 $$\begin{gathered} {\rm N}_{{\overset{\lower0.5em\hbox{$\smash{\scriptscriptstyle\frown}$}}{\phi } }} \left[ {\overset{\lower0.5em\hbox{$\smash{\scriptscriptstyle\frown}$}}{\phi } (\eta ;\zeta ),\overset{\lower0.5em\hbox{$\smash{\scriptscriptstyle\frown}$}}{f} (\eta ;\zeta ),\overset{\lower0.5em\hbox{$\smash{\scriptscriptstyle\frown}$}}{\theta } (\eta ;\zeta )} \right] = \left( {1 + 2\gamma \eta } \right)\overset{\lower0.5em\hbox{$\smash{\scriptscriptstyle\frown}$}}{\phi }_{\eta \eta } + Sc\overset{\lower0.5em\hbox{$\smash{\scriptscriptstyle\frown}$}}{f} \overset{\lower0.5em\hbox{$\smash{\scriptscriptstyle\frown}$}}{\phi }_{\eta } + 2\gamma \overset{\lower0.5em\hbox{$\smash{\scriptscriptstyle\frown}$}}{\phi }_{\eta } \hfill \\ \,\,\,\,\,\,\,\,\,\,\,\,\,\,\,\,\,\,\,\,\,\,\,\,\,\,\,\,\,\,\,\,\,\,\,\,\,\,\,\,\,\,\,\,\,\,\,\,\,\,\,\,\,\,\,\,\,\,\,\,\,\,\,\,\,\,\, + \frac{{N_{t} }}{{N_{b} }}\left[ {\left( {1 + 2\gamma \eta } \right)\overset{\lower0.5em\hbox{$\smash{\scriptscriptstyle\frown}$}}{\theta }_{\eta \eta } + 2\gamma \overset{\lower0.5em\hbox{$\smash{\scriptscriptstyle\frown}$}}{\theta }_{\eta } } \right] \hfill \\ \end{gathered}$$

For Eqns. (7–9) the 0th-order system is written as

20 $$(1 - \zeta )L_{{\overset{\lower0.5em\hbox{$\smash{\scriptscriptstyle\frown}$}}{f} }} \left[ {\overset{\lower0.5em\hbox{$\smash{\scriptscriptstyle\frown}$}}{f} (\eta ;\zeta ) - \overset{\lower0.5em\hbox{$\smash{\scriptscriptstyle\frown}$}}{f}_{0} (\eta )} \right] = p\hbar_{{\overset{\lower0.5em\hbox{$\smash{\scriptscriptstyle\frown}$}}{f} }} {\rm N}_{{\overset{\lower0.5em\hbox{$\smash{\scriptscriptstyle\frown}$}}{f} }} \left[ {\overset{\lower0.5em\hbox{$\smash{\scriptscriptstyle\frown}$}}{f} (\eta ;\zeta ),\overset{\lower0.5em\hbox{$\smash{\scriptscriptstyle\frown}$}}{\theta } (\eta ;\zeta ),(\eta ;\zeta )} \right]$$

21 $$(1 - \zeta ) \, L_{{\overset{\lower0.5em\hbox{$\smash{\scriptscriptstyle\frown}$}}{\theta } }} \left[ {\overset{\lower0.5em\hbox{$\smash{\scriptscriptstyle\frown}$}}{\theta } (\eta ;\zeta ) - \overset{\lower0.5em\hbox{$\smash{\scriptscriptstyle\frown}$}}{\theta }_{0} (\eta )} \right] = \,\,\,p\,\hbar_{{\overset{\lower0.5em\hbox{$\smash{\scriptscriptstyle\frown}$}}{\theta } }} \,{\rm N}_{{\overset{\lower0.5em\hbox{$\smash{\scriptscriptstyle\frown}$}}{\theta } }} \,\,\left[ {\overset{\lower0.5em\hbox{$\smash{\scriptscriptstyle\frown}$}}{f} (\eta ;\zeta ),\,\,\overset{\lower0.5em\hbox{$\smash{\scriptscriptstyle\frown}$}}{\theta } (\eta ;\zeta ),\overset{\lower0.5em\hbox{$\smash{\scriptscriptstyle\frown}$}}{\phi } (\eta ,\zeta )} \right]$$

22 $$(1 - \zeta ) \, L_{{\overset{\lower0.5em\hbox{$\smash{\scriptscriptstyle\frown}$}}{\phi } }} \left[ {\overset{\lower0.5em\hbox{$\smash{\scriptscriptstyle\frown}$}}{\phi } (\eta ;\zeta )\,\,\, - \,\,\overset{\lower0.5em\hbox{$\smash{\scriptscriptstyle\frown}$}}{\phi }_{0} (\eta )} \right] = p\,\,\hbar_{{\overset{\lower0.5em\hbox{$\smash{\scriptscriptstyle\frown}$}}{\phi } }} \,\,{\rm N}_{{\overset{\lower0.5em\hbox{$\smash{\scriptscriptstyle\frown}$}}{\phi } }} \,\,\,\left[ {\overset{\lower0.5em\hbox{$\smash{\scriptscriptstyle\frown}$}}{\phi } (\eta ;\zeta )\,,\,\,\overset{\lower0.5em\hbox{$\smash{\scriptscriptstyle\frown}$}}{f} (\eta ;\zeta ),\,\,\overset{\lower0.5em\hbox{$\smash{\scriptscriptstyle\frown}$}}{\theta } \,(\eta ;\zeta )} \right]$$

For subjected BCs we have

23 $$\begin{gathered} \left. {\overset{\lower0.5em\hbox{$\smash{\scriptscriptstyle\frown}$}}{f} (\eta ;\zeta )} \right|_{\eta = 0} = 0, \, \,\,\,\,\,\,\,\,\,\,\left. {\frac{{\partial \overset{\lower0.5em\hbox{$\smash{\scriptscriptstyle\frown}$}}{f} (\eta ;\zeta )}}{\partial \eta }} \right|_{\eta = 0} = 1,\left. {\,\,\,\,\,\,\,\,\,\,\,\,\,\,\,\,\,\frac{{\partial \overset{\lower0.5em\hbox{$\smash{\scriptscriptstyle\frown}$}}{\theta } (\eta ;\zeta )}}{\partial \eta }} \right|_{\eta = 0} = - \gamma_{1} \left[ {1 - \left. {\overset{\lower0.5em\hbox{$\smash{\scriptscriptstyle\frown}$}}{\theta } (\eta ;\zeta )} \right|_{\eta = 0} } \right], \hfill \\ \left. {\frac{{\partial \overset{\lower0.5em\hbox{$\smash{\scriptscriptstyle\frown}$}}{\phi } (\eta ;\zeta )}}{\partial \eta }} \right|_{\eta = 0} = - \gamma_{2} \left[ {1 - \left. {\overset{\lower0.5em\hbox{$\smash{\scriptscriptstyle\frown}$}}{\phi } (\eta ;\zeta )} \right|_{\eta = 0} } \right], \hfill \\ \left. {\frac{{\partial \overset{\lower0.5em\hbox{$\smash{\scriptscriptstyle\frown}$}}{f} (\eta ;\zeta )}}{\partial \eta }} \right|_{\eta = \infty } = 0,\left. {\,\,\,\,\,\,\,\,\,\,\,\overset{\lower0.5em\hbox{$\smash{\scriptscriptstyle\frown}$}}{\theta } (\eta ;\zeta )} \right|_{\eta = \infty } = 0,\left. {\,\,\,\,\,\,\,\,\,\overset{\lower0.5em\hbox{$\smash{\scriptscriptstyle\frown}$}}{\phi } (\eta ;\zeta )} \right|_{\eta = \infty } = \,\,\,\,0 \hfill \\ \end{gathered}$$

When $\zeta = 0{\text{ and }}\zeta = 1$ we have (here $\zeta \in [0,1]$[0,1])

24 $$\overset{\lower0.5em\hbox{$\smash{\scriptscriptstyle\frown}$}}{f} (\eta ;1) = \overset{\lower0.5em\hbox{$\smash{\scriptscriptstyle\frown}$}}{f} (\eta ),\overset{\lower0.5em\hbox{$\smash{\scriptscriptstyle\frown}$}}{\theta } (\eta ;1) = \overset{\lower0.5em\hbox{$\smash{\scriptscriptstyle\frown}$}}{\theta } (\eta ){ ,}\overset{\lower0.5em\hbox{$\smash{\scriptscriptstyle\frown}$}}{\phi } (\eta ;1) = \overset{\lower0.5em\hbox{$\smash{\scriptscriptstyle\frown}$}}{\phi } (\eta ),$$

Taylor’s expansion for $$\overset{\lower0.5em\hbox{$\smash{\scriptscriptstyle\frown}$}}{f} (\eta ;\zeta ) \, ,\overset{\lower0.5em\hbox{$\smash{\scriptscriptstyle\frown}$}}{\theta } (\eta ;\zeta )$$ and $$\overset{\lower0.5em\hbox{$\smash{\scriptscriptstyle\frown}$}}{\phi } (\eta \,\,;\zeta )$$ about $$\zeta = 0$$

25 $$\begin{gathered} \overset{\lower0.5em\hbox{$\smash{\scriptscriptstyle\frown}$}}{f} (\eta ;\zeta ) \, = \, \overset{\lower0.5em\hbox{$\smash{\scriptscriptstyle\frown}$}}{f}_{0} (\eta ) + \sum\nolimits_{n = 1}^{\infty } {\overset{\lower0.5em\hbox{$\smash{\scriptscriptstyle\frown}$}}{f}_{n} (\eta )\zeta^{n} } \hfill \\ \overset{\lower0.5em\hbox{$\smash{\scriptscriptstyle\frown}$}}{\theta } (\eta ;\zeta ) \, = \, \overset{\lower0.5em\hbox{$\smash{\scriptscriptstyle\frown}$}}{\theta }_{0} (\eta ) + \sum\nolimits_{n = 1}^{\infty } {\overset{\lower0.5em\hbox{$\smash{\scriptscriptstyle\frown}$}}{\theta }_{n} (\eta )\zeta^{n} } \hfill \\ \overset{\lower0.5em\hbox{$\smash{\scriptscriptstyle\frown}$}}{\phi } (\eta ;\zeta ) \, = \, \overset{\lower0.5em\hbox{$\smash{\scriptscriptstyle\frown}$}}{\phi }_{0} (\eta ) + \sum\nolimits_{n = 1}^{\infty } {\overset{\lower0.5em\hbox{$\smash{\scriptscriptstyle\frown}$}}{\phi }_{n} (\eta )\zeta^{n} } \hfill \\ \end{gathered}$$

26 $$\overset{\lower0.5em\hbox{$\smash{\scriptscriptstyle\frown}$}}{f}_{n} (\eta ) \, = \left. {\frac{1}{n!}\frac{{\partial \overset{\lower0.5em\hbox{$\smash{\scriptscriptstyle\frown}$}}{f} (\eta ;\zeta )}}{\partial \eta }} \right|_{p = 0} ,\overset{\lower0.5em\hbox{$\smash{\scriptscriptstyle\frown}$}}{\theta }_{n} (\eta ) \, = \left. {\frac{1}{n!}\frac{{\partial \overset{\lower0.5em\hbox{$\smash{\scriptscriptstyle\frown}$}}{\theta } (\eta ;\zeta )}}{\partial \eta }} \right|_{p = 0} ,\overset{\lower0.5em\hbox{$\smash{\scriptscriptstyle\frown}$}}{\phi }_{n} (\eta ) \, = \left. {\frac{1}{n!}\frac{{\partial \overset{\lower0.5em\hbox{$\smash{\scriptscriptstyle\frown}$}}{\phi } (\eta ;\zeta )}}{\partial \eta }} \right|_{p = 0} \, {.}$$

With BCs as

27 $$\begin{gathered} {\overset{\lower0.5em\hbox{$\smash{\scriptscriptstyle\frown}$}}{f} }\left( 0 \right) = 0,\overset{\lower0.5em\hbox{$\smash{\scriptscriptstyle\frown}$}}{f^{\prime}} \left( 0 \right) = 1,\overset{\lower0.5em\hbox{$\smash{\scriptscriptstyle\frown}$}}{\theta }^{\prime}\left( 0 \right) = - \gamma_{1} \left[ {1 - \overset{\lower0.5em\hbox{$\smash{\scriptscriptstyle\frown}$}}{\theta } \left( 0 \right)} \right],\overset{\lower0.5em\hbox{$\smash{\scriptscriptstyle\frown}$}}{\phi }^{\prime}\left( 0 \right) = - \gamma_{2} \left[ {1 - \overset{\lower0.5em\hbox{$\smash{\scriptscriptstyle\frown}$}}{\phi } \left( 0 \right)} \right]\,\, \hfill \\ \overset{\lower0.5em\hbox{$\smash{\scriptscriptstyle\frown}$}}{f^{\prime}} \left( \infty \right) = 0,\overset{\lower0.5em\hbox{$\smash{\scriptscriptstyle\frown}$}}{g} \left( \infty \right) = 0,\overset{\lower0.5em\hbox{$\smash{\scriptscriptstyle\frown}$}}{\theta } \left( \infty \right) = 0,\overset{\lower0.5em\hbox{$\smash{\scriptscriptstyle\frown}$}}{\phi } \left( \infty \right) = 0, \hfill \\ \end{gathered}$$

Now

28 $$\begin{gathered} \Re_{n}^{{\overset{\lower0.5em\hbox{$\smash{\scriptscriptstyle\frown}$}}{f} }} \left( \eta \right) = 2\left( {1 + \frac{1}{\lambda }} \right)f^{\prime\prime\prime}_{n - 1} \,\,\, + \,\,\,2\gamma \overset{\lower0.5em\hbox{$\smash{\scriptscriptstyle\frown}$}}{f^{\prime\prime}}_{n - 1} \,\,\, + \,\,\,2\sum\limits_{j = 0}^{w - 1} {\overset{\lower0.5em\hbox{$\smash{\scriptscriptstyle\frown}$}}{f}_{w - 1 - j} \overset{\lower0.5em\hbox{$\smash{\scriptscriptstyle\frown}$}}{f^{\prime\prime}}_{j} } - f^{\prime 2}_{n - 1} \hfill \\ - \beta_{1} {\text{Re}} \left( {\frac{{\sum\limits_{j = 0}^{w - 1} {\overset{\lower0.5em\hbox{$\smash{\scriptscriptstyle\frown}$}}{f}_{w - 1 - j}^{2} \overset{\lower0.5em\hbox{$\smash{\scriptscriptstyle\frown}$}}{f^{\prime\prime}}_{j} } }}{\eta }\,\,\, + \,\,2\sum\limits_{j = 0}^{w - 1} {\overset{\lower0.5em\hbox{$\smash{\scriptscriptstyle\frown}$}}{f}_{w - 1 - j}^{2} \overset{\lower0.5em\hbox{$\smash{\scriptscriptstyle\frown}$}}{f^{\prime\prime\prime}}_{j} } \,\,\, - \,4\sum\limits_{j = 0}^{w - 1} {\overset{\lower0.5em\hbox{$\smash{\scriptscriptstyle\frown}$}}{f}_{w - 2 - j}^{2} \overset{\lower0.5em\hbox{$\smash{\scriptscriptstyle\frown}$}}{f}^{\prime}_{w - 1 - j} \overset{\lower0.5em\hbox{$\smash{\scriptscriptstyle\frown}$}}{f^{\prime\prime}}_{j} } } \right) \hfill \\ - \,M\,\,\,\left( {\overset{\lower0.5em\hbox{$\smash{\scriptscriptstyle\frown}$}}{f}^{\prime}_{n - 1} \,\,\, - \,\,\,\beta_{1} \sum\limits_{j = 0}^{w - 1} {\overset{\lower0.5em\hbox{$\smash{\scriptscriptstyle\frown}$}}{f}_{w - 1 - j} \,\,\,\overset{\lower0.5em\hbox{$\smash{\scriptscriptstyle\frown}$}}{f^{\prime\prime}}_{j} } } \right)\,\,\, + \,\,\,\lambda \left( {\overset{\lower0.5em\hbox{$\smash{\scriptscriptstyle\frown}$}}{\theta }^{\prime} + N_{r} \overset{\lower0.5em\hbox{$\smash{\scriptscriptstyle\frown}$}}{\phi } } \right) \hfill \\ \end{gathered}$$

29 $$\begin{gathered} \Re_{n}^{{\overset{\lower0.5em\hbox{$\smash{\scriptscriptstyle\frown}$}}{\theta } }} (\eta ) = \left( {2\gamma \eta \,\,\, + \,\,1} \right)\overset{\lower0.5em\hbox{$\smash{\scriptscriptstyle\frown}$}}{\theta^{\prime\prime}}_{n - 1} + \,\,\Pr \sum\limits_{j = 0}^{w - 1} {\overset{\lower0.5em\hbox{$\smash{\scriptscriptstyle\frown}$}}{\theta }^{\prime}_{w - 1 - j} } \hat{f}_{j} + {\text{PrN}}_{b} \,\,\left( {2\gamma \eta \,\,\, + \,\,1} \right)_{0} \sum\limits_{j = 0}^{w - 1} {\overset{\lower0.5em\hbox{$\smash{\scriptscriptstyle\frown}$}}{\phi^{\prime}}_{w - 1 - j} } \overset{\lower0.5em\hbox{$\smash{\scriptscriptstyle\frown}$}}{\theta }^{\prime}_{j} \hfill \\ + \Pr M\left( {Ec_{1} \overset{\lower0.5em\hbox{$\smash{\scriptscriptstyle\frown}$}}{f}^{\prime 2}_{n - 1} \,\,\, + \,\,Ec_{2} \overset{\lower0.5em\hbox{$\smash{\scriptscriptstyle\frown}$}}{g}_{n - 1} } \right)\,\, + \,\,\,\delta \Pr {\text{Re}} \overset{\lower0.5em\hbox{$\smash{\scriptscriptstyle\frown}$}}{\theta }_{n - 1} + \Pr N_{t} \left( {2\gamma \eta \,\,\, + \,\,1} \right)\theta^{\prime 2}_{n - 1} \,\, \hfill \\ \end{gathered}$$

30 $$\Re \,_{n}^{{\overset{\lower0.5em\hbox{$\smash{\scriptscriptstyle\frown}$}}{\phi } }} \,\left( \eta \right) = \left( {2\gamma \eta \,\,\, + \,\,\,1} \right)\,\,\,\overset{\lower0.5em\hbox{$\smash{\scriptscriptstyle\frown}$}}{\phi }^{\prime\prime}_{n - 1} + Sc\sum\limits_{j = 0}^{w - 1} {\overset{\lower0.5em\hbox{$\smash{\scriptscriptstyle\frown}$}}{f}_{w - 1 - j} \overset{\lower0.5em\hbox{$\smash{\scriptscriptstyle\frown}$}}{\phi }^{\prime}_{j} } + 2\,\,\gamma \,\overset{\lower0.5em\hbox{$\smash{\scriptscriptstyle\frown}$}}{\theta^{\prime}}_{n - 1} + \frac{{N_{t} }}{{N_{b} }}\left[ {\left( {2\gamma \eta \,\,\, + \,\,\,1} \right)\overset{\lower0.5em\hbox{$\smash{\scriptscriptstyle\frown}$}}{\theta }^{\prime\prime}_{n - 1} + 2\gamma \overset{\lower0.5em\hbox{$\smash{\scriptscriptstyle\frown}$}}{\theta }^{\prime}_{n - 1} } \right]$$

31 $${\text{While}}\;\;\;\chi_{n} = \left\{ \begin{gathered} 0,{\text{ if }}\zeta \le {1} \hfill \\ 1,{\text{ if }}\zeta > {1}{\text{.}} \hfill \\ \end{gathered} \right.$$

### Convergence analysis

**Figure 2:**
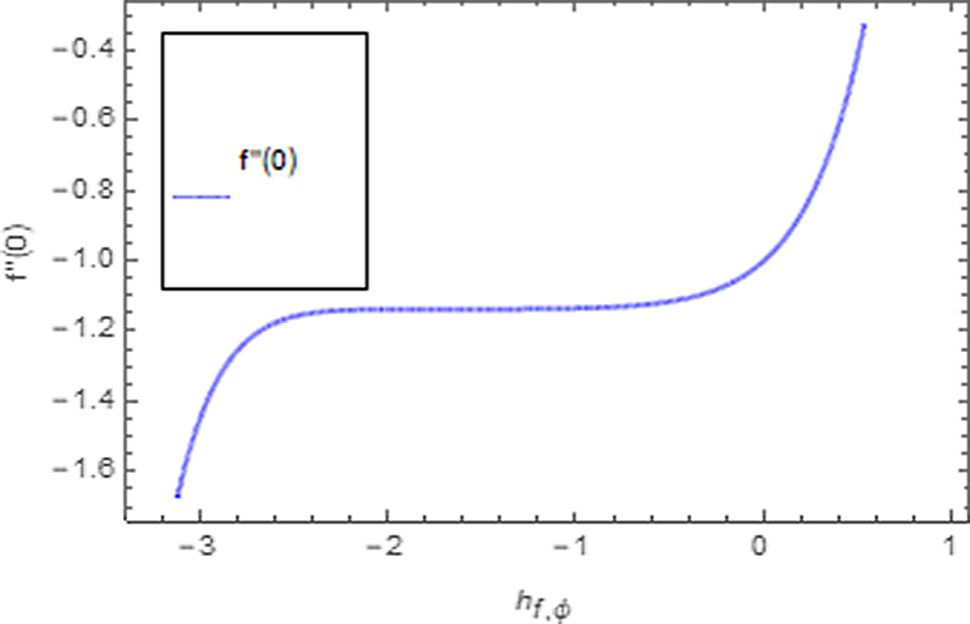
$$h -$$ curve for $$f^{\prime}\left( \eta \right)$$.

**Figure 3:**
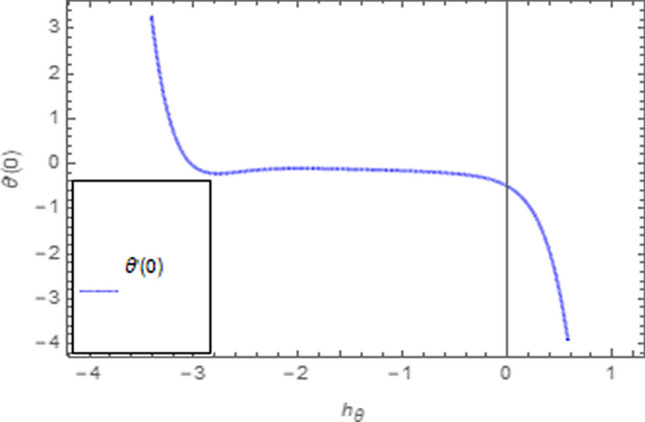
$$h -$$ curve for $$\theta \left( \eta \right)$$.

**Figure 4:**
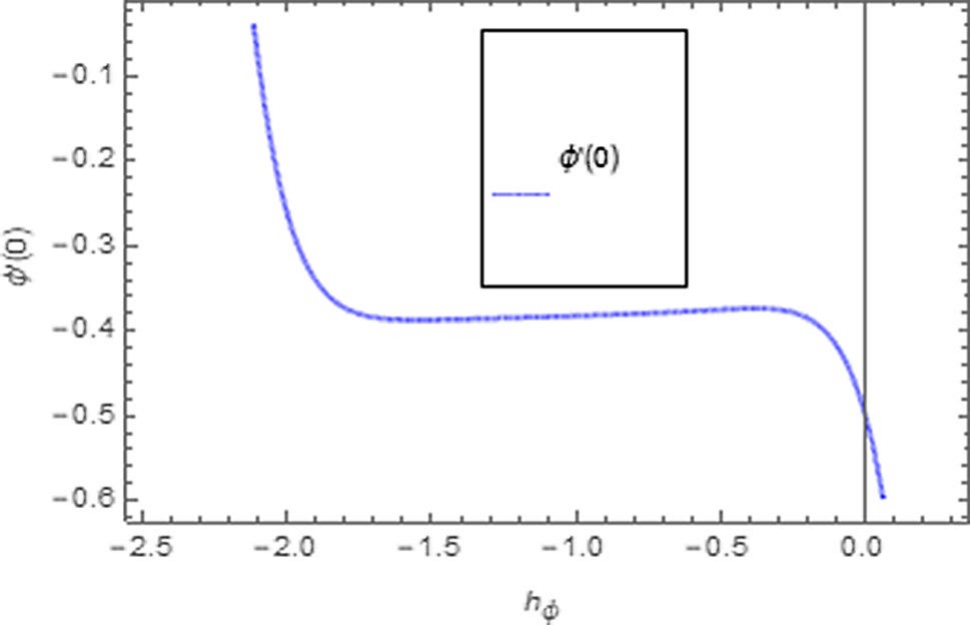
$$h -$$ curve for $$\phi \left( \eta \right)$$.

**Figure 5:**
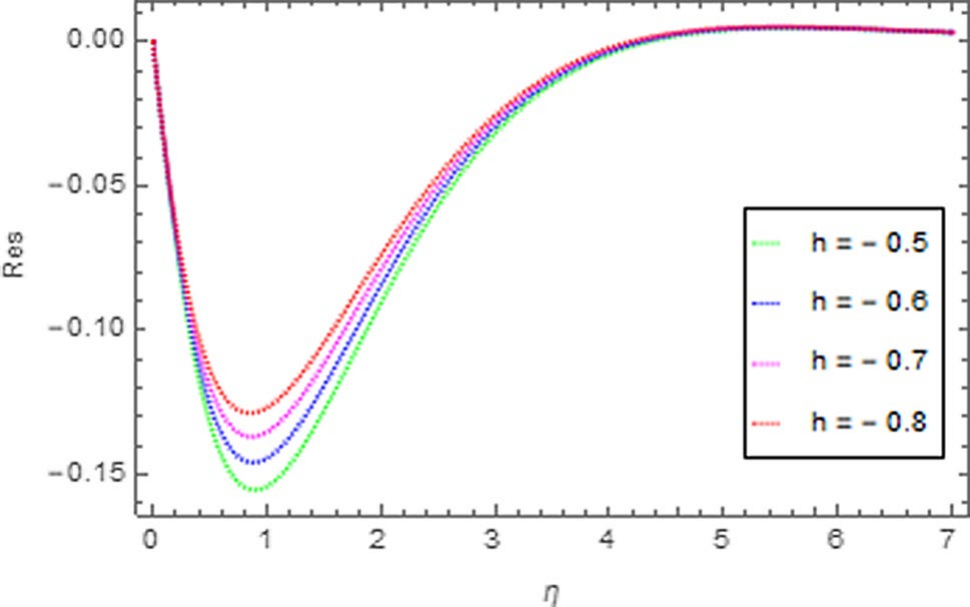
Residual errors sketch for solution using 10th order HAM approximation.

To use HAM we need to find out solutions in series for velocity, temperature and concentration functions. In determination of these solutions, the auxiliary parameters $\rlap{--} h_{f} ,\,\rlap{--} h_{\theta }$ and $\rlap{--} h_{\phi }$ are encountered which are dependable for convergence of solution. To check the region of validity for these parameters,we have constructed h-curves at 10th order of approximation (see Figs. 2, 3, 4). From these figures we have noticed that the range for convergence is $- 3.5 \le \rlap{--} h_{f} \le 0,\, - 3.0\, \le \rlap{--} h_{\theta } \le 0.5\,\,and\,\, - 2.0 \le \rlap{--} h_{\phi } \le 0.\,1.$ Moreover, Fig. 5 shows the residual errors of the HAM solution for various values of $h$.

## Results and discussion

This work describes the mixed convection flow for Maxwell nanofluid with transfer of thermal energy over a stretching and rotating cylinder. The behaviors of different substantial parameters have been examined and discussed graphically. Numerical tables are also constructed to discuss impact of these parameters upon various profiles of flow system.

### Flow characteristics

**Figure 6:**
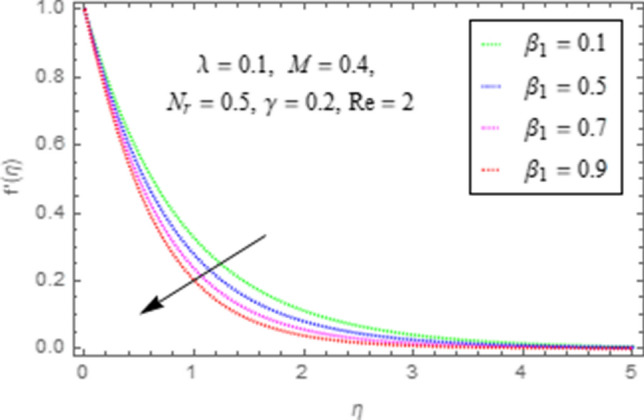
Impact of $\beta_{1}$ upon $f^{\prime}\left( \eta \right)$.

**Figure 7:**
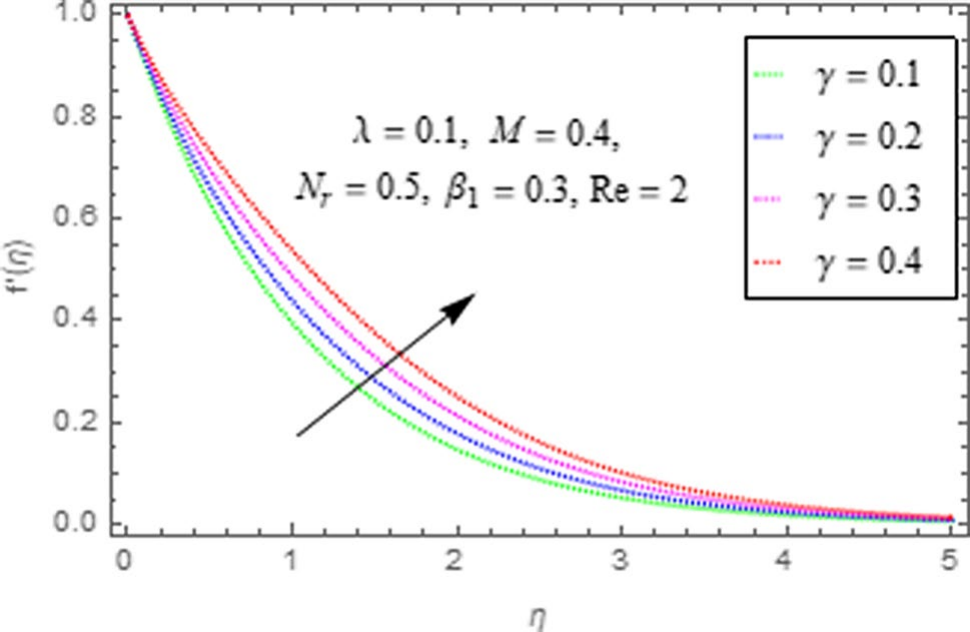
Impact of $\gamma$ upon $f^{\prime}\left( \eta \right)$.

**Figure 8:**
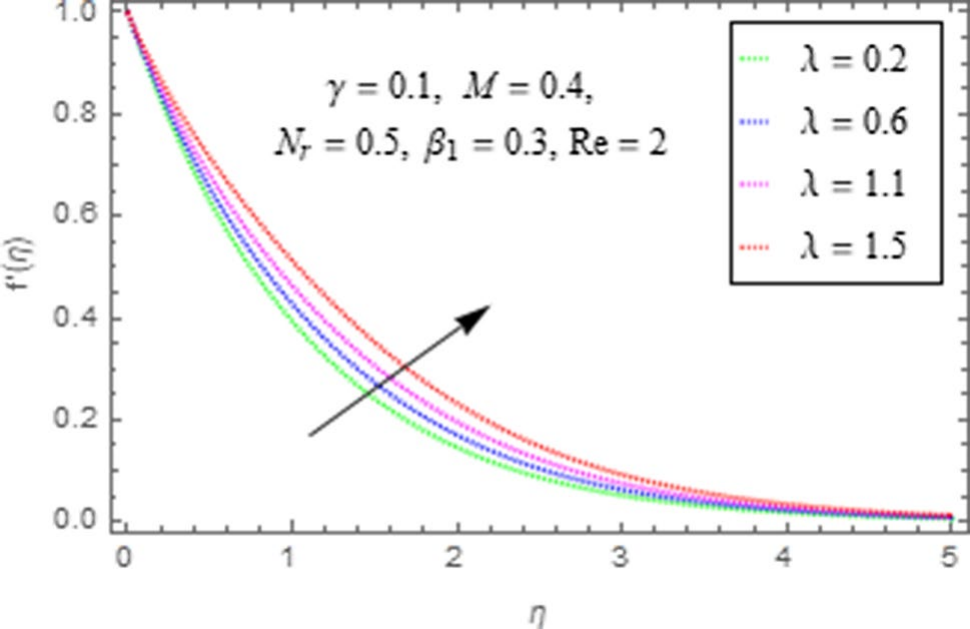
Impact of $\lambda$ upon $f^{\prime}\left( \eta \right)$.

**Figure 9:**
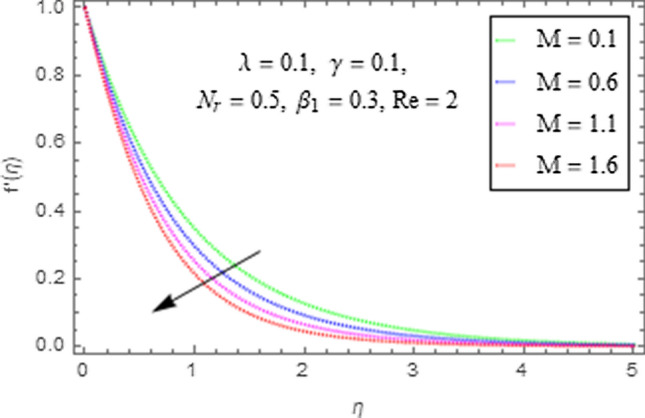
Impact of $M$ upon $f^{\prime}\left( \eta \right)$.

**Figure 10:**
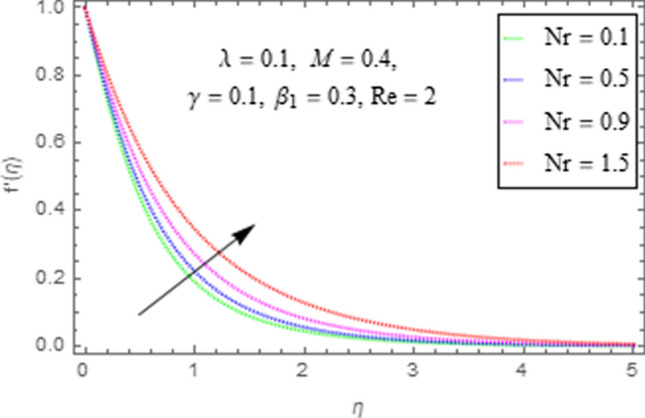
Impact of $Nr$ upon $f^{\prime}\left( \eta \right)$.

**Figure 11:**
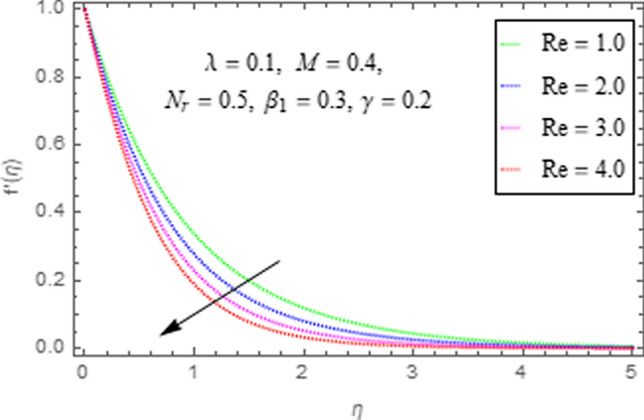
Impact of ${\text{Re}}$ upon $f^{\prime}\left( \eta \right)$.

This subsection describes the impact of various physical parameters upon flow characteristics. These parameters include $\beta_{1} =$ Maxwell parameter, $\gamma =$ curvature parameter, $\lambda =$ mixed convection parameter, $M =$ Hartman number, $N_{r} =$ Buoyancy ratio and ${\text{Re}} =$ Reynolds number as shown in Figs. 6, 7, 8, 9, 10, 11. From Fig. 6 we see that velocity reduces with larger values of $\beta_{1} .$ Actually enhancing values of $\beta_{1}$ boost up the stress relaxation phenomenon, as a result of which flow characteristics of nanofluid declines. Figure 7 depicts impact of $\gamma$ upon velocity. Since for $\gamma$ to be higher, the radius of cylinder augments, ultimately fluid flow enhances. Figure 8 describes impact of $\lambda$ on fluid flow. Here it is obvious that increasing values of $\lambda$, increase fluid flow. Actually with augmentation in mixed convection parameter, buoyancy forces increase as a result of this physical phenomenon there is a corresponding growth in flow characteristics of nanofluid. Impact of Hartman number upon velocity depicts in Fig. 9. Since when intensity of magnetic field increases it generates a resistive force in opposite direction of flow. Therefore, higher values of $M$ decline velocity distribution of nanofluid. Impact of Buoyancy ratio upon flow of fluid is depicted in Fig. 10. It is understood that growth in $N_{r}$ results in an augmentation in velocity distribution. Impact of Reynolds number on velocity profile portrays in Fig. 11. Thisfigure describes that growing values of $({\text{Re}} )\,$ results in decliningof flow field. Physically it can be interpreted as, with growing values of Reynolds number the inertial force of nanoparticles increases. Now since inertial force is an opposing agent for fluid flow of nanoparticles, hence motion of the fluid decreases.

### Thermal characteristics

**Figure 12:**
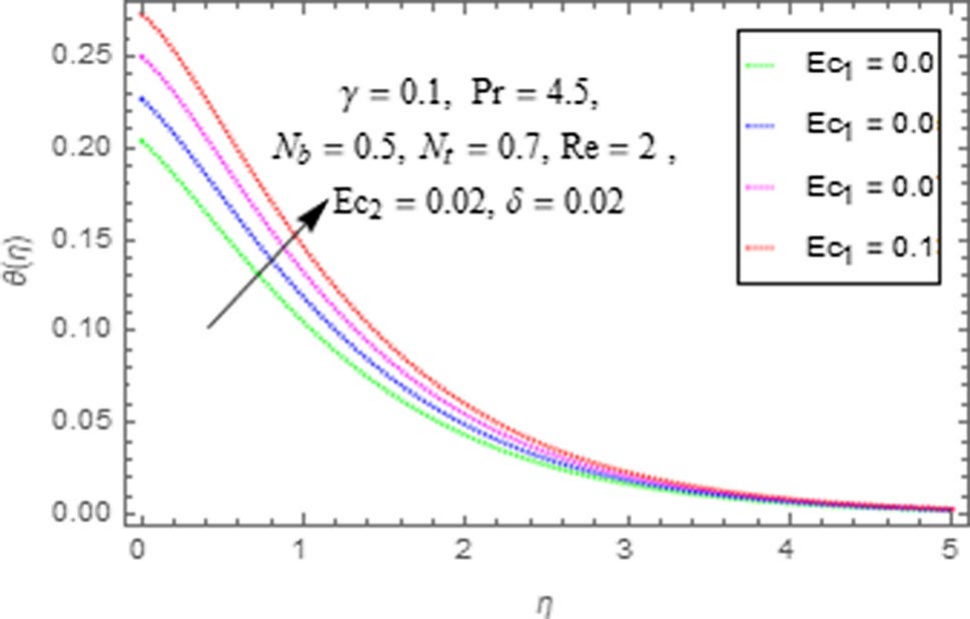
Impact of $Ec_{1}$ upon $\theta \left( \eta \right)$.

**Figure 13:**
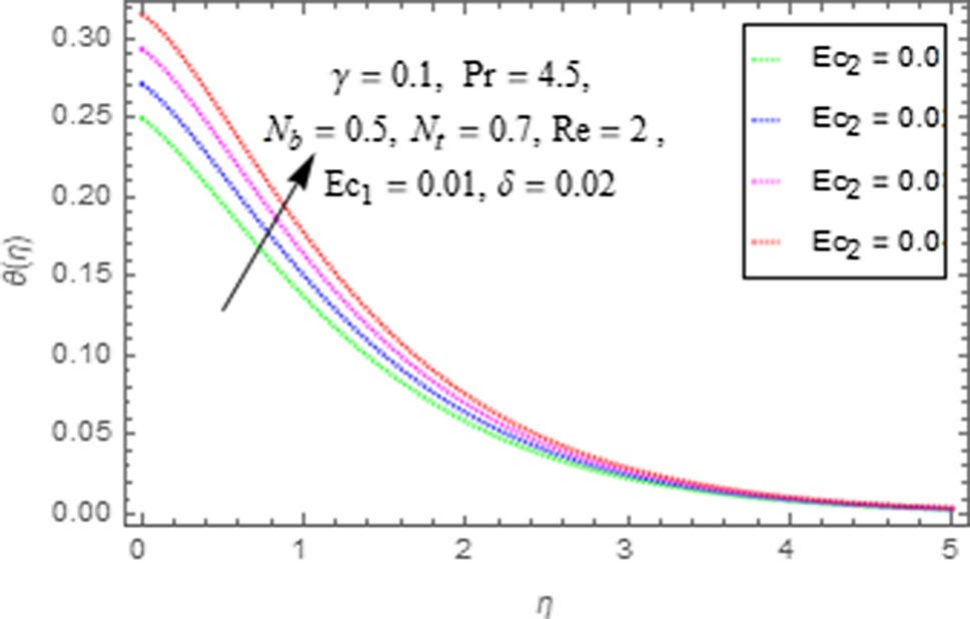
Impact of $Ec_{2}$ upon $\theta \left( \eta \right)$.

**Figure 14:**
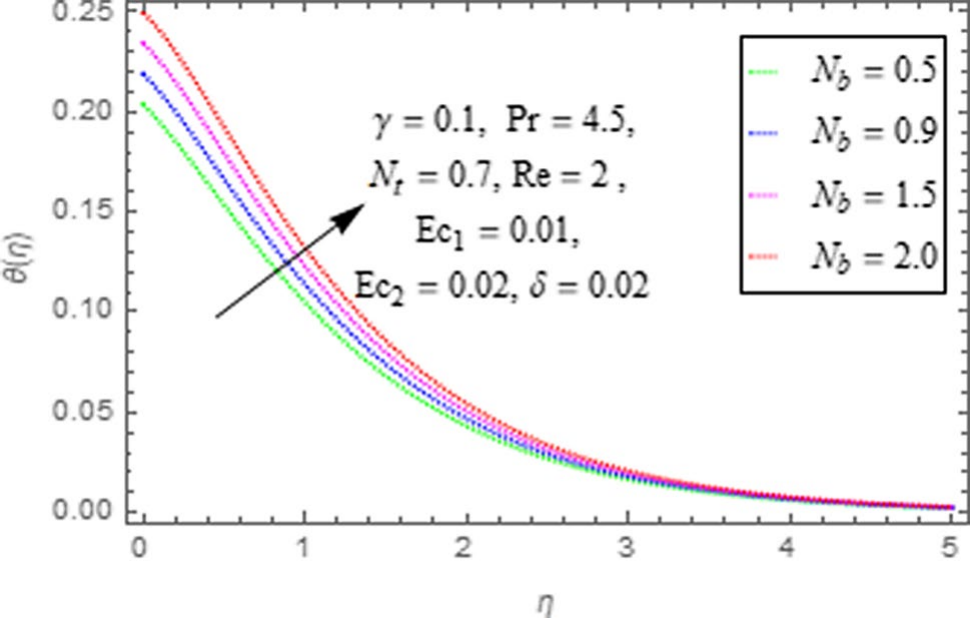
Impact of $N_{b}$ upon $\theta \left( \eta \right)$.

**Figure 15:**
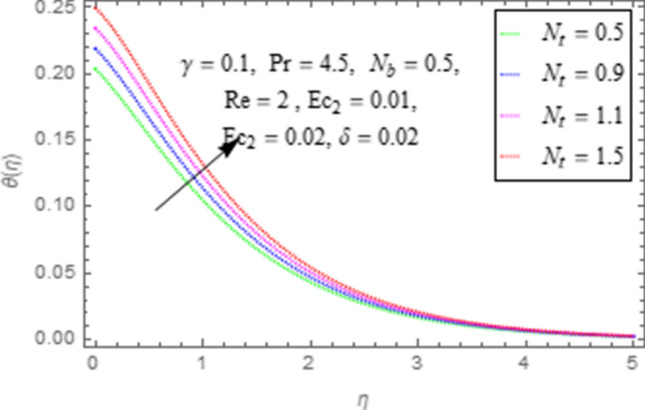
Impact of $N_{t}$ upon $\theta \left( \eta \right)$.

**Figure 16:**
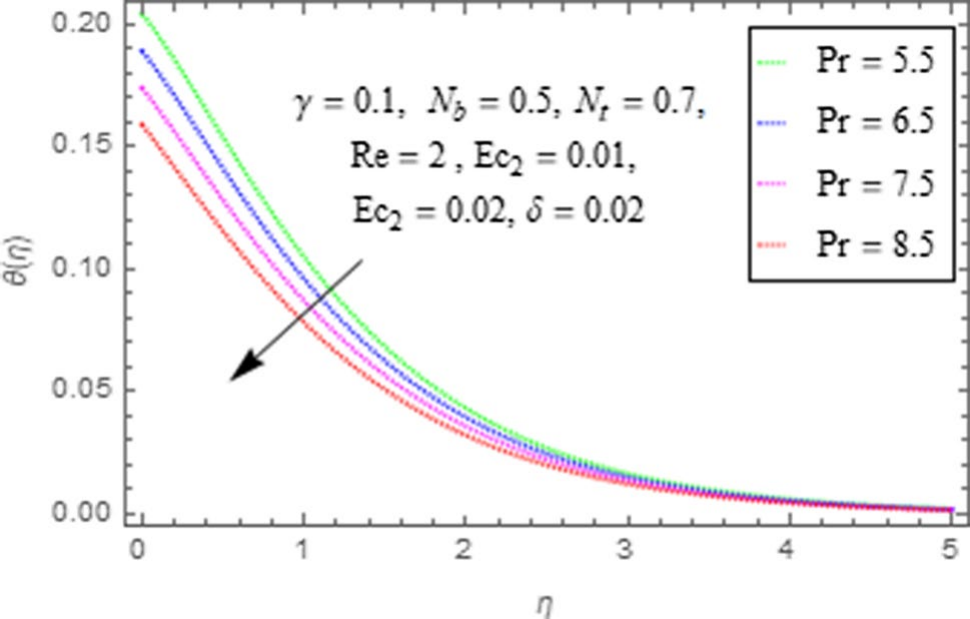
Impact of $\Pr$ upon $\theta \left( \eta \right)$.

**Figure 17:**
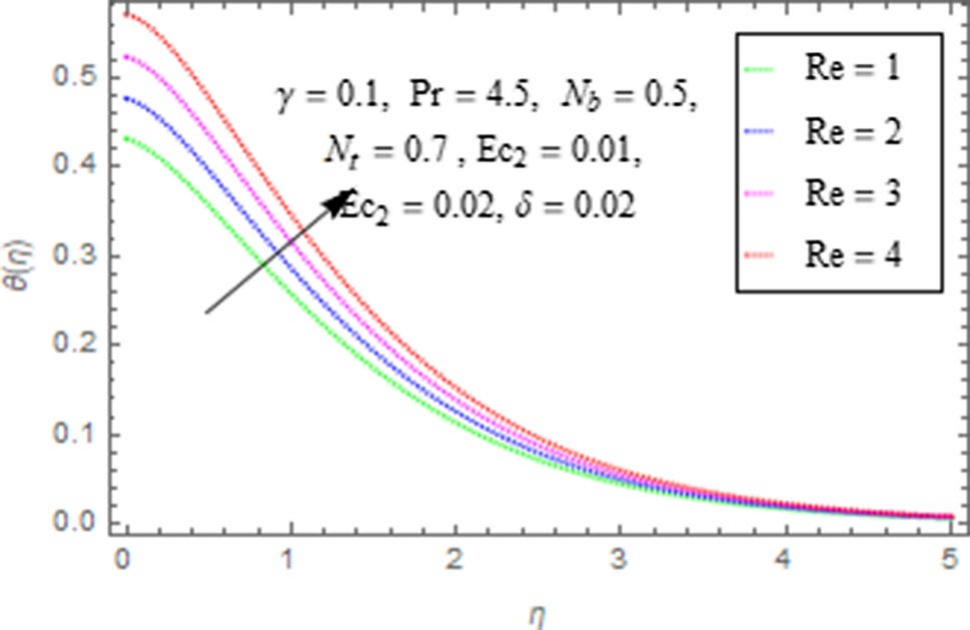
Impact of ${\text{Re}}$ upon $\theta \left( \eta \right)$.

**Figure 18:**
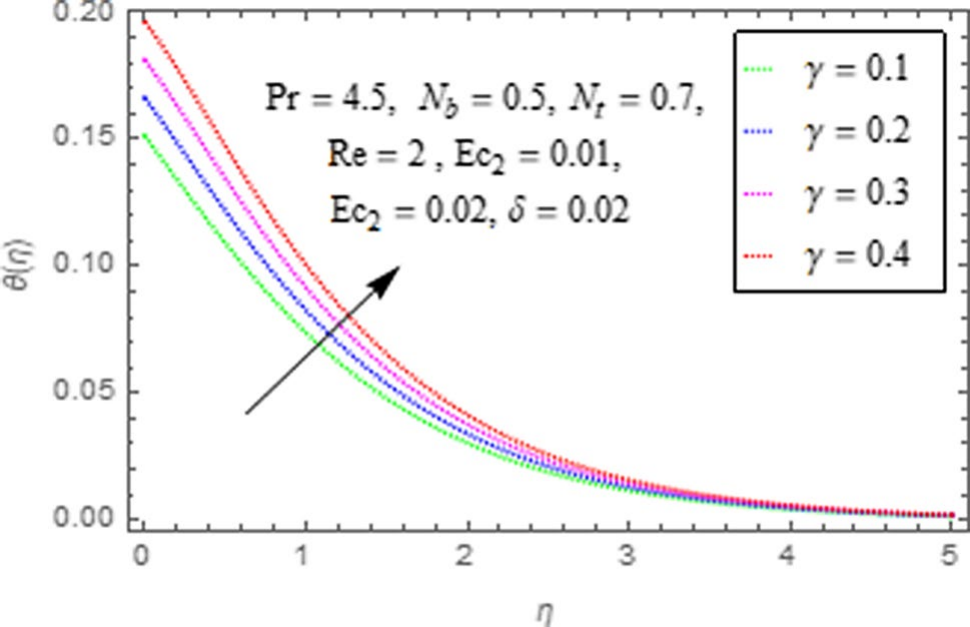
Impact of $\gamma$ upon $\theta \left( \eta \right)$.

**Figure 19:**
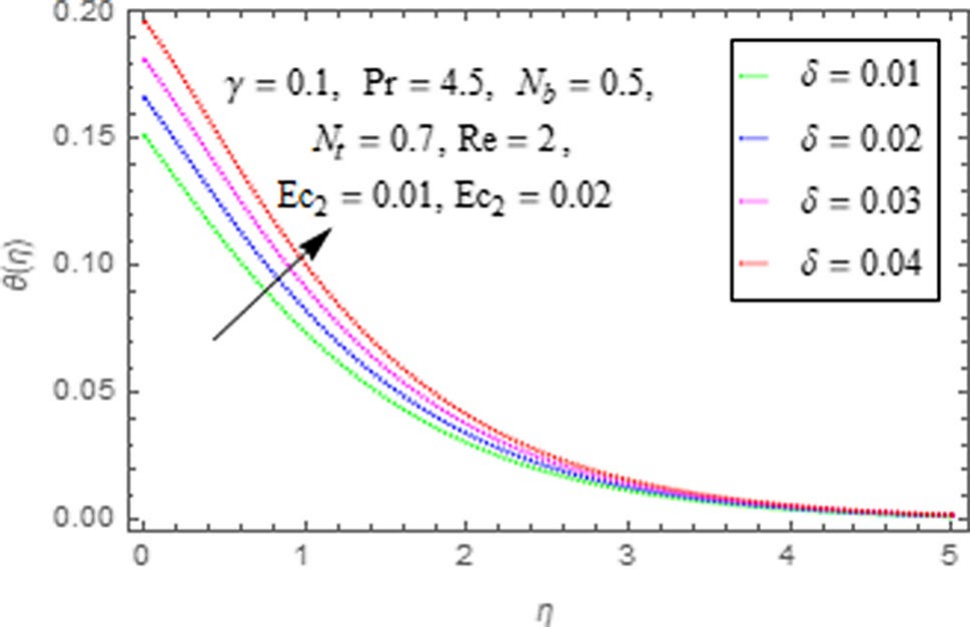
Impact of $\delta$ upon $\theta \left( \eta \right)$.

**Figure 20:**
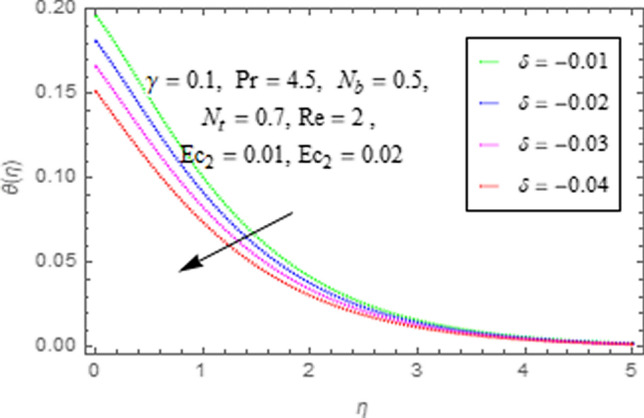
Impact of $(\delta )$ upon $\theta \left( \eta \right)$.

This subsection describes impact of $Ec_{1} =$ Eckert number for cylinder’s stretching, $Ec_{2} =$ Eckert number for cylinder’s rotation, $N_{b} =$ Brownian motion parameter, $N_{t} =$ Thermophoresis parameter, $\Pr =$ Prandtl number, ${\text{Re}} =$ Reynolds number, $\gamma =$ curvature parameter and $\delta =$ Heat generation/absorption parameter upon thermal characteristics of nanofluid as highlighted in Figs. 12, 13, 14, 15, 16, 17, 18, 19, 20. The impact of Eckert numbers (both used for stretching and rotation of cylinder) upon temperature is depicted in Figs. 12, 13. From these figures it is observed that due to enhancement in Eckert number there is a growth in thermal energy transportation of Maxwell nanofluid. Since $Ec$ (ratio of kinetic energy to thermal energy transport driving force) represents Joule heating effects.Therefore, augmentation in $Ec$ enhances temperature of nanofluid. Figure 14 depicts effect of $N_{b}$ upon temperature. Since for growth in $N_{b}$ there is a corresponding augmentation in random motion ofnanoparticles. This increase in random motion results in increasing the collision of nanoparticles, due to which kinetic energy is transformed to heat. Therefore, for augmentation in $N_{b}$ there is an increase in temperature distribution of fluid. Impact of Thermophoresis parameter $N_{t}$ upon temperature depicts in Fig. 15. Since $N_{t} = \frac{{\tau D_{T} \Delta T}}{{T_{\infty } \upsilon }}$, so increase in thermophoresis parameter means augmentation in temperature gradient. So for higher values of $N_{t}$ we have corresponding growth in temperature. Impact of Prandtl number upon temperature is discussed in Fig. 16. We see that temperature decreases with augmentation inPrandtl number. Actually when Prandtl number increases, then mass as well as thermal diffusivity of nanoparticles reduceand henceits temperature reduces. Figure 17 depicts impact of Reynolds number over temperature distribution. Since increasing values of $Re$ results in a reduction of convection force of nanoparticles and hence there is an increase in thermal characteristics of nanoparticles. Figure 18 depicts impact of curvature parameter $\gamma$ on $\theta \left( \eta \right)$. We see that temperature augments with growing values of curvature parameter. Impact of $\delta$ upon temperature depicts in Figs. 19, 20 both for $\delta > 0,\,\,\delta < 0$. Since for heat source $\delta > 0$ we see that some additional heat is produced, that enhances heat transport properties of flow system, hence temperature of system enhances in this case as shown in Fig. 19. Moreover, a reverse impact is observed for heat sink $\delta < 0$. Actually for $\delta < 0$ the transport characteristics of flow system reduces that ultimately reduces temperature of nanofluid as shown in Fig. 20.

### Concentration characteristics

**Figure 21:**
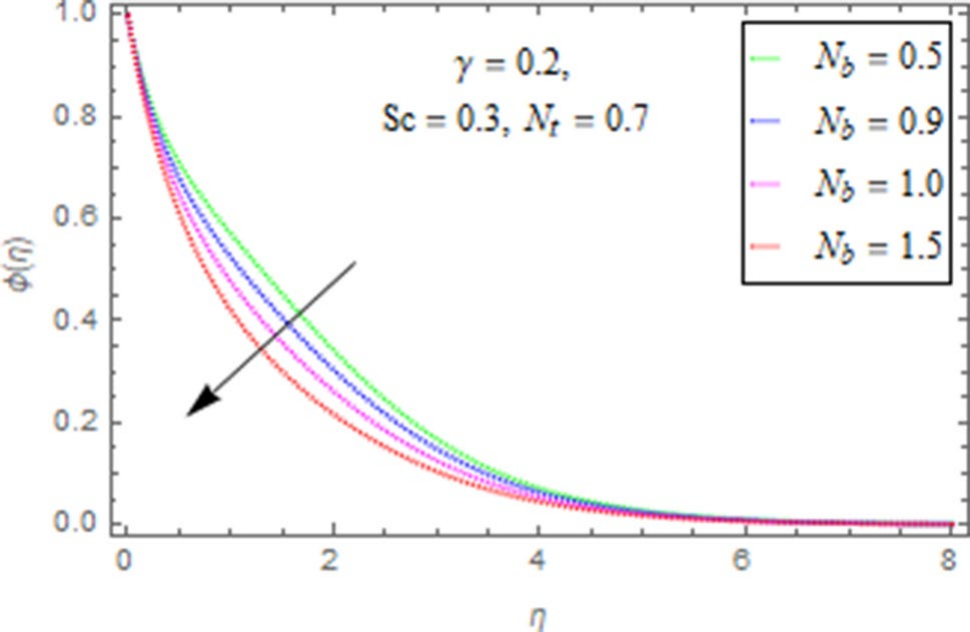
Impact of $N_{b}$ upon $\phi \left( \eta \right)$.

**Figure 22:**
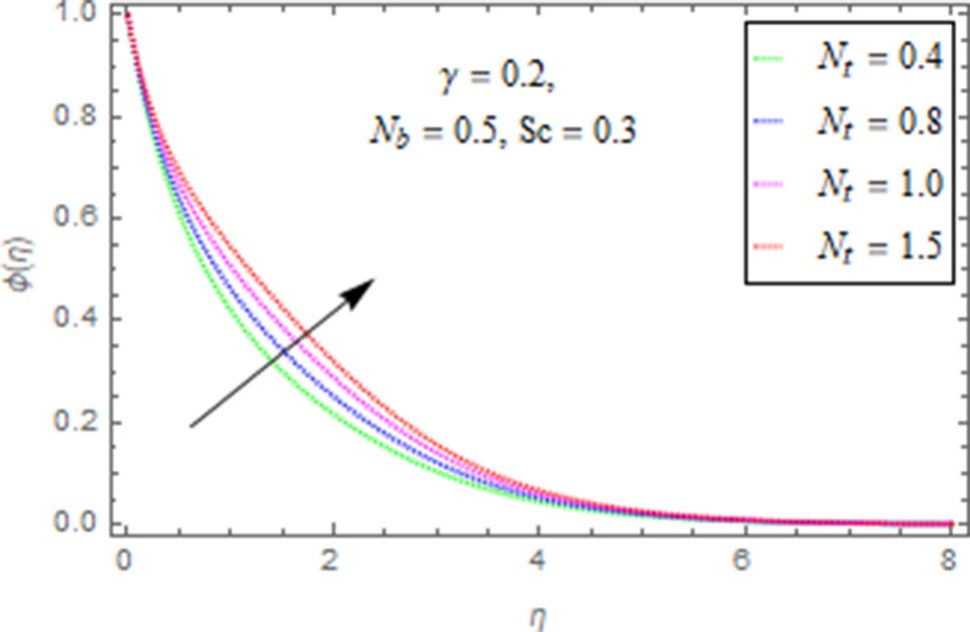
Impact of $N_{t}$ upon $\phi \left( \eta \right)$.

**Figure 23:**
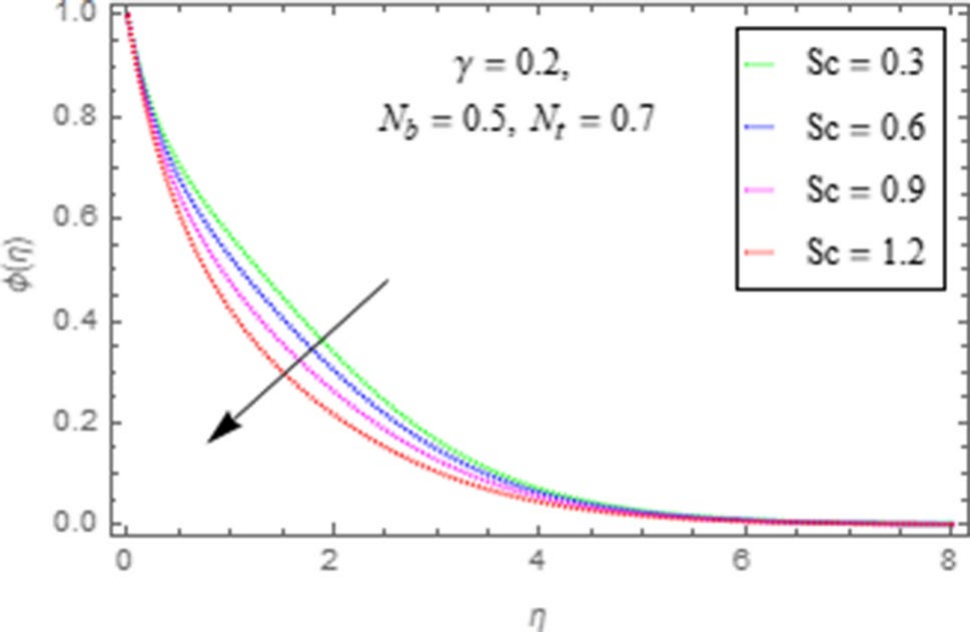
Impact of $Sc$ upon $\phi \left( \eta \right)$.

This subsection describes impact of $N_{b} =$ Brownian motion parameter, $N_{t} =$ Thermophoresis parameter and $Sc =$ Schmidt number upon concentration distribution $\phi \left( \eta \right)$ as given in Figs. 21, 22, 23. Figure 21 portrays effect of $N_{b}$ upon $\phi \left( \eta \right)$. It is obvious from this figure that concentration of nanofluid reduces for higher values of $N_{b}$. Actually Brownian motion is haphazard motion of nanoparticles (which are suspended in base fluid) and is more influenced by fast moving molecules. Figure 22 describes that with higher values of $N_{t}$, the concentration of fluid grows up. In fact when $N_{t}$ increases then temperature differences between wall and free surface also increases that ultimately enhances concentration of nanofluid. Fi 23 depicts impact of Schmidt number upon concentration distribution. From this figure it is determined that enhancing values of $Sc$ reduces concentration of nanofluid. In fact, when $Sc$ increases then molecular/mass diffusivity of fluid reduces that ultimately reduces the concentration of nanoparticles as shown in Fig. 23.

### Table discussion

The numerical values for different substantial parameters upon velocity profile, temperature gradient and concentration gradient are evaluated in Tables 1, 2 and 3 respectively. Moreover, a comparison is also carried out for validation of current work with the results as available in literature^46^. It is observed from this comparison that our result is in good conformity with the filed values which are presented in Table 4.

**Table 1:** Impact of various substantial parameters over velocity profiles $f(\eta )$ and $f^{\prime}(\eta )$.

${\text{Re}}$	$\gamma$	$\beta 1$	$M$	$f(\eta )$	$f^{\prime}(\eta )$
0.1	0.23	0.31	0.25	$0.8313793$	$0.1306459$
0.2				$0.8258029$	$0.1280804$
0.3				$0.8202604$	$0.1255435$
	0.1			$0.8263999$	$0.1238384$
	0.2			$0.8302507$	$0.1306459$
	0.3			$0.8339640$	$0.1370183$
		0.1		$0.8398284$	$0.1365566$
		0.2		$0.8357958$	$0.1345714$
		0.3		$0.8317799$	$0.1326024$
			0.1	$0.8573742$	$0.1440031$
			0.2	$0.8399046$	$0.1361867$
			0.3	$0.8229916$	$0.1287101$

**Table 2:** Impact of various substantial parameters over temperature $\theta (\eta )$.

$\delta$	$\gamma$	$\Pr$	$Ec1$	$Ec2$	$M$	$\theta (\eta )$
0.3	0.23	0.31	0.25	0.29	0.15	$0.0911114$
0.4						$0.0918603$
0.5						$0.0926147$
	0.3					$0.0938488$
	0.4					$0.0976639$
	0.5					$0.1013696$
		0.3				$0.0907708$
		0.4				$0.0941896$
		0.5				$0.0976367$
			0.3			$0.0917239$
			0.4			$0.0929489$
			0.5			$0.0941740$
				0.3		$0.0911394$
				0.4		$0.0914196$
				0.5		$0.0917006$
					0.3	$0.0948719$
					0.4	$0.0973467$
					0.5	$0.0997969$

**Table 3:** Impact of over concentration $\phi (\eta )$ various substantial parameters.

$\gamma$	$Sc$	$\phi (\eta )$
0.5	0.45	$0.0347714$
0.6		$0.0292947$
0.7		$0.0240052$
0.37	0.5	$0.0417823$
	0.6	$0.0409927$
	0.7	$0.0402080$

**Table 4:** Comparison between present result and Ref.^46^ for Nusselt and Sherwood numbers at different values of substantial parameters $\gamma ,\,\,N_{b} ,\,\,N_{t} ,\,\,\gamma_{1} \,\,and\,\,\gamma_{2}$.

$\gamma$	$N_{b}$	$N_{t}$	$\gamma_{1}$	$\gamma_{2}$	$Nu_{z} ({\text{Re}}_{z} )^{ - 1/2}$		$Sh_{z} ({\text{Re}}_{z} )^{ - 1/2}$	
					Ref.^46^	Present work	Ref.^46^	Present work
0.1	0.1	0.1	0.2	0.7	0.15571	$0.155721$	0.28856	0.288572
0.2					0.15712	$0.157130$	0.29877	0.298784
0.3					0.15839	$0.158402$	0.30942	0.309439
0.1	0.2				0.15459	$0.154603$	0.31332	0.313337
	0.3				0.15352	$0.153531$	0.32173	0.321746
	0.4				0.15243	$0.152440$	0.32602	0.326039
	0.1	0.2			0.15553	$0.155543$	0.24168	0.241691
		0.3			0.15532	$0.155334$	0.19622	0.196230
		0.4			0.15513	$0.155142$	0.15235	0.152367
		0.1	0.5		0.29332	$0.293337$	0.25090	0.250919
			0.7		0.35292	$0.352936$	0.23510	0.235116
			0.9		0.39792	$0.397938$	0.22329	0.223391
			0.2	0.5	0.15578	$0.155791$	0.24135	0.241365
				0.8	0.15569	$0.155691$	0.30742	0.307436
				1.0	0.15563	$0.155640$	0.33836	0.338374

## Conclusions

This work describes the mixed convection flow for Maxwell nanofluid with transfer of thermal energy over a stretching and rotating cylinder in the presence of Joule heating and Heat generation/absorption. The modeled problem is solved by HAM.The behaviors of different substantial parameters have been examined and discussed graphically. Tables are also constructed to see numerically the impact of different substantial parameters upon flow characteristics.Moreover, a comparison is also carried out for validation of current work with the results as available in literature^46^. It is observed from this comparison that our result is in good agreement with the filed values. After detailed study of the article the following observations have been noticed:

- Enhancing values of Maxwell parameter boost up the stress relaxation phenomenon, as a result of which flow characteristics of nanofluid reduces.
- Increase in mixed convection parameter increases buoyancy forces due to this physical phenomenon, flow characteristics of nanofluid also increase.
- Rise in intensity of magnetic field, generates Lorentz forces that produces a resistive force in reverse direction of flow field and ultimately declines velocity distribution of nanofluid.
- It is observed that due to enhancement in Eckert number there is an increase in thermal energy transportation of Maxwell nanofluid.
- Increase in Brownian motion of nanoparticles, converts kinetic energy into heat energy, that ultimately increases temperature.
- Increase in thermophoresis parameter, increases temperature gradient that result in augmentation of nanoparticles temperature.
- With augmentation in Prandtl number, the mass as well as thermal diffusivities of nanoparticles reduce,as an outcome of which warmth of fluid reduces.
- For heat source $\delta > 0$, some additional heat is produced, that enhances heat transport properties of flow system and hence temperature of system enhances in this case. A reverse impact has seen for heat sink $\delta < 0$.
- When thermophoresis parameter increases then temperature differences between wall and free surface also increases that ultimately enhances concentration of nanofluid.
- For elevated values of Schmidt numbert hemolecular/mass diffusivity of fluid reduces, that ultimately reduces the concentration of nanoparticles.

## Symbols

$u,\,\,w$: Axial and radial velocity components $\left( {{\text{ms}}^{ - 1} } \right)$
$T_{\infty }$: Ambient fluid temperature $\left( {\text{K}} \right)$
$C_{\infty }$: Ambient fluid concentration
$D_{B}$: Brownian diffusion coefficient $\left( {{\text{m}}^{2} {\text{s}}^{ - 1} } \right)$
$N_{b}$: Brownian motion parameter
$$N_{r}$$: Buoyancy ratio
$$l$$: Characteristic length $$\left( {\text{m}} \right)$$
$$T$$: Dimensional temperature $$\left( {\text{K}} \right)$$
$$C$$: Dimensional concentration
$Ec_{1}$: Eckert number for cylinder’s stretching
$Ec_{2}$: Eckert number for cylinder’s rotation
$$M$$: Hartman number
$T_{f}$: Heated fluid temperature $\left( {\text{K}} \right)$
$C_{f}$: Heated fluid concentration
$Nu_{z}$: Local Nusselt number
$\,\,Sh_{z}$: Local Sherwood number
${\text{Re}}_{z}$: Local Reynolds number
$B_{0}$: Magnetic field strength $\left( {{\text{NmA}}^{ - 1} } \right)$
$k_{m}$: Mass transfer coefficient
$D_{m}$: Molecular diffusivity of species concentration
$$\Pr$$: Prandtl number
$$U_{0}$$: Reference velocity $$\left( {{\text{ms}}^{ - 1} } \right)$$
$${\text{Re}}$$: Reynolds number
$$Sc$$: Schmidt number
$h_{m}$: Surface mass flux $\left( {{\text{W}}\;{\text{m}}^{ - 2} } \right)$
$q_{w}$: Surface heat flux $\left( {{\text{W}}\;{\text{m}}^{ - 2} } \right)$
$$k$$: Thermal conductivity $$\left( {{\text{W}}\;{\text{K}}^{ - 1} {\text{m}}^{ - 1} } \right)$$
$D_{T}$: Thermophoretic diffusion coefficient $\left( {{\text{m}}^{2} {\text{s}}^{ - 1} } \right)$
$$N_{t}$$: Thermophoresis parameter

## Greek symbols

$$\gamma$$: Curvature parameter
$\beta_{T}$: Coefficient of thermal expansion
$\gamma_{2}$: Concentration Biot number
$\phi$: Dimensionless fluid concentration
$$\,\theta$$: Dimensionless temperature
$$\rho$$: Density $$\left( {{\text{kg}}\;{\text{m}}^{ - 3} } \right)$$
$\delta$: Heat generation/absorption parameter
$$\upsilon$$: Kinematic viscosity $$\left( {{\text{m}}^{2} {\text{s}}^{ - 1} } \right)$$
$$\beta_{1}$$: Maxwell parameter
$\lambda$: Mixed convection parameter
$$\eta$$: Similarity variable
$$\alpha_{1}$$: Thermal diffusivity $$\left( {{\text{m}}\;{\text{s}}^{ - 1} } \right)$$
$\gamma_{1}$: Thermal Biot number

## Superscript

ʹ: Prime denotes derivative w. r. t. $\eta$

## Abbreviations

HAM: Homotopy analysis method
ODEs: Ordinary differential equations
RK-4: Runge–Kutta method of order four
MHD: Magnetohydrodynamics
PDEs: Partial differential equations
